# Multiple Calcifying Pseudoneoplasms of the Neuraxis

**DOI:** 10.7759/cureus.1044

**Published:** 2017-02-21

**Authors:** Leonardo B Brasiliense, Dennis W Dickson, Raouf E Nakhleh, Rabih G Tawk, Robert Wharen

**Affiliations:** 1 Neurosurgery, Division of Neurosurgery, University of Arizona, Tucson, AZ; 2 Pathology, Mayo Clinic, Jacksonville, FL; 3 Department of Neurosurgery, Mayo Clinic, Jacksonville, FL; 4 Neurosurgery / Neuro Oncology Program, Mayo Clinic, Jacksonville, FL

**Keywords:** benign, calcified pseudoneoplasm, supratentorial, tumor

## Abstract

Calcifying pseudoneoplasms of the neuraxis (CAPNONs) are extremely rare tumors that are frequently misdiagnosed and overlooked by clinicians. To date, only 40 intracranial lesions have been reported, and in all instances, they were found as a solitary calcified mass. To our knowledge, the current case report is the first to illustrate the development of multiple intraaxial CAPNONs and shed more light on the origin of these lesions.

We discuss the case of a 67-year-old woman who presented with a six-year history of recurrent seizures. Magnetic resonance imaging revealed two similar heterogeneous intracranial masses in the ventral midbrain and left frontal white matter with indications of more aggressive behavior in the supratentorial tumor. The lesion was resected, and histopathological analysis showed tissue containing nodules of chondromyxoid material with a coarsely fibrillar matrix and focal alveolar pattern. Palisading cells were noted around the edges as well as dystrophic calcifications and osseous metaplasia, consistent with CAPNON. Interestingly, the patient had a previous history of multiple brain abscesses and a mycotic aneurysm. At her four-month follow-up visit, the patient remained seizure-free and there were no indications of residual tumor or recurrence.

In contrast to previous reports, intracranial CAPNONs may manifest as multiple lesions and clinicians should include these tumors in the differential diagnosis of intra-axial calcified masses. The previous history of brain abscesses raises the suspicion of an abnormal proliferative process following an insult to the brain tissue as the underlying origin of these lesions.

## Introduction

Calcifying pseudoneoplasms of the neuraxis (CAPNON), also known as fibro-osseous lesions, are extremely rare tumors that can be located from the extremities to the central nervous system [1]. Despite the lack of information on their natural history, these lesions are considered benign, non-invasive tumors that cause morbidity due to regional mass effect rather than neurovascular invasion [2]. The origin of CAPNONs, however, remains controversial. It has been proposed that these tumors may arise from a reactive proliferative process [3], a metaplastic transformation [4], or a neoplastic development [5]. Since the microscopic features of CAPNONs were first described in 1978, only 40 intracranial lesions have been reported in the literature [6]. In all instances, intracranial CAPNONs have been found as a solitary mass and have been associated with nonspecific imaging findings suggestive of a well-circumscribed, densely calcified lesion. To our knowledge, the current case report is the first to illustrate the development of multiple intra-axial CAPNONs, both supratentorial and infratentorial.

## Case presentation

**Figure 1:**
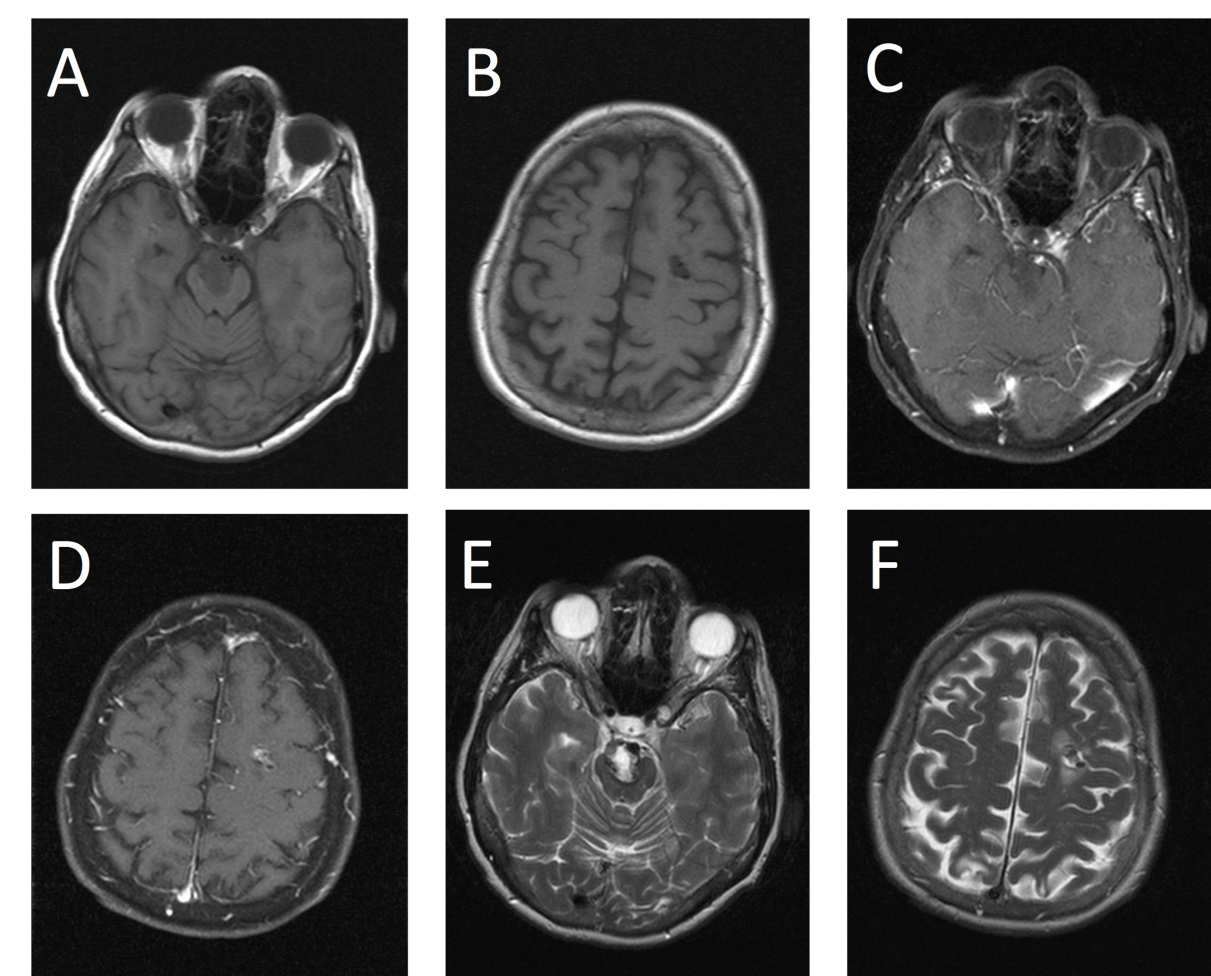
MRI demonstrating multiple CAPNONs Magnetic resonance imaging (MRI). T1-weighted images pre-contrast depicting two hypointense masses located in the ventral brainstem (A) and left frontal lobe (B). T1-weighted images post-contrast showing no contrast enhancement in the brainstem lesion (C) and heterogeneous enhancement on the left frontal lesion (D). T2-weighted images demonstrate T2 hyperintensity (E) and edema around the left frontal mass (F).

A 67-year-old woman presented with a six-year history of recurrent focal seizures with impaired consciousness suggesting a left hemispheric origin. The patient had a recent increase in the frequency of seizures with poor response to medical treatment. Magnetic resonance imaging (MRI) at the onset of seizures revealed multiple heterogeneous intracranial lesions located in the ventral midbrain and in the left frontal white matter. The MRI features of the lesions were fairly similar, demonstrating hypointensity on T1- and T2-weighted images. The lesion in the left frontal lobe showed moderate contrast enhancement as well as a mild adjacent hyperintense signal on T2-weighted images. Minimal to no contrast enhancement was observed around the lesion in the brainstem (Figure 1).

**Figure 2:**
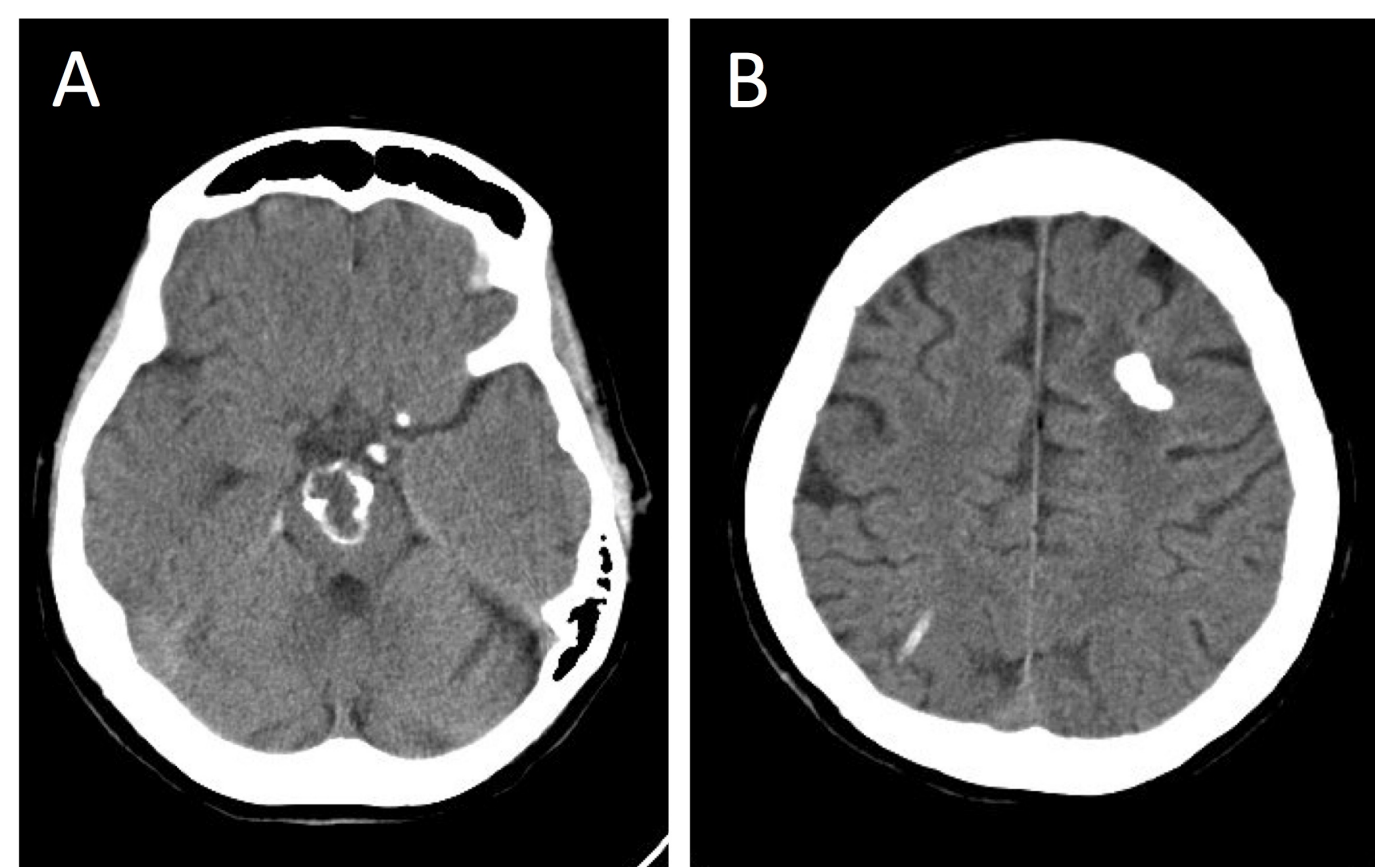
Computed tomography showing multiple calcified lesions Computed tomography (CT) images. Axial brain window images depicting hyperdense, calcified lesions in the brainstem (A) and left frontal lobe (B).

Computed tomography (CT) demonstrated dense calcifications in the supratentorial lesion and peripheral calcifications in the brainstem mass (Figure 2).

**Figure 3:**
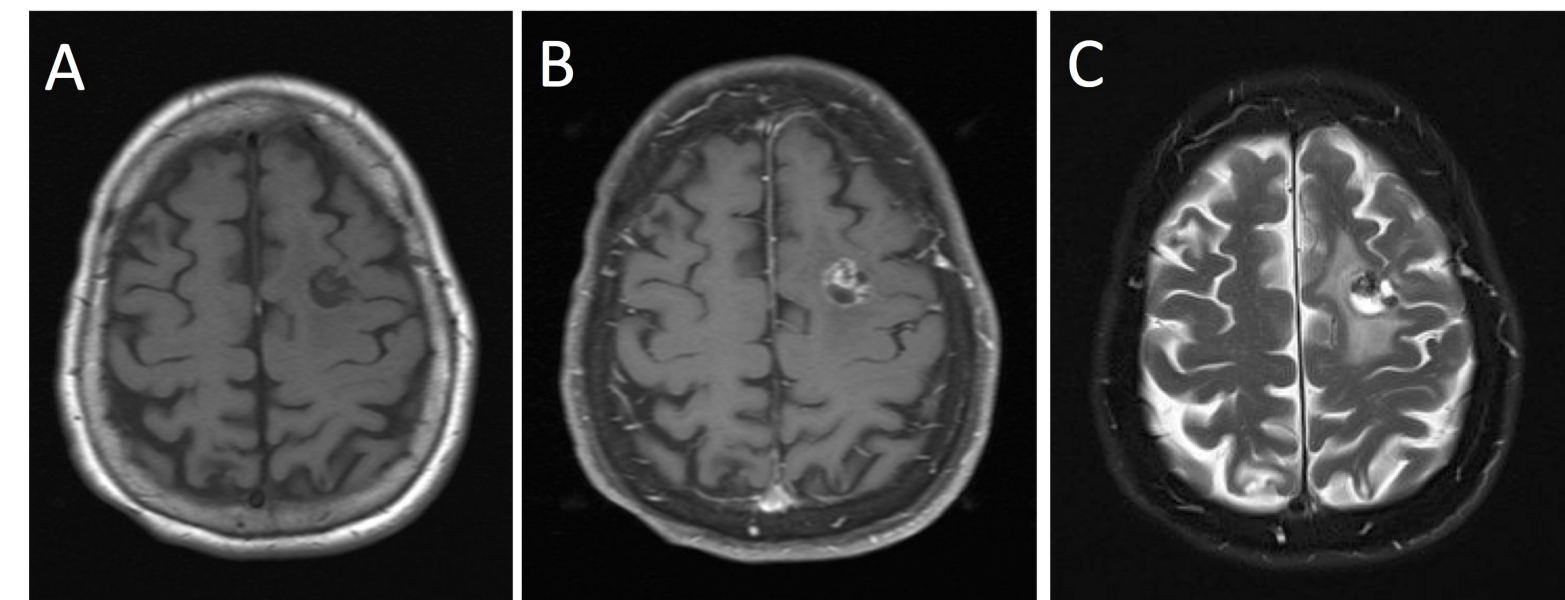
MRI evidence of lesion growth Magnetic resonance imaging (MRI). T1-weighted image pre-contrast (A) and post-contrast (B) demonstrating interval growth of the left frontal lesion. T2-weighted image showing increased area of T2 hyperintensity around the left frontal mass (C).

Of note, the patient had a remote history of infection with multiple brain abscesses and a mycotic aneurysm of the right posterior cerebral artery. Reportedly, the infection occurred 30 years before the onset of seizures and was attributed to rheumatic heart disease and infective endocarditis. Her physical examination showed a partial right third cranial nerve palsy, which was the sequelae of her aneurysmal subarachnoid hemorrhage, and a new right pronator drift. An MRI scan performed six months later showed interval growth of the left frontal mass and an increased area of T2-weighted hyperintensity in the adjacent brain (Figure 3).

Based on imaging characteristics of the lesions, the differential diagnosis included multiple cavernous malformations, calcified meningiomas, intracranial infections, and other less likely diagnoses, including primary osseous and chondroid malignant tumors (e.g. chondrosarcoma). After reviewing the management options, the patient elected to proceed with left frontal tumor resection, as it was likely responsible for her worsening symptoms. Informed patient consent was obtained for her treatment.

### Intraoperative characteristics

The lesion was approached and resected through a left frontal craniotomy. The arachnoid was noticeably thickened over the mass. The lesion was extremely firm and near entirely calcified with an irregular, tanned/pink appearance as well as minimal internal vascularity. The lesion was dissected from the surrounding brain tissue and “en bloc” resection was achieved with no complications. Postoperative MRI demonstrated complete resection.

###  Pathological findings

**Figure 4:**
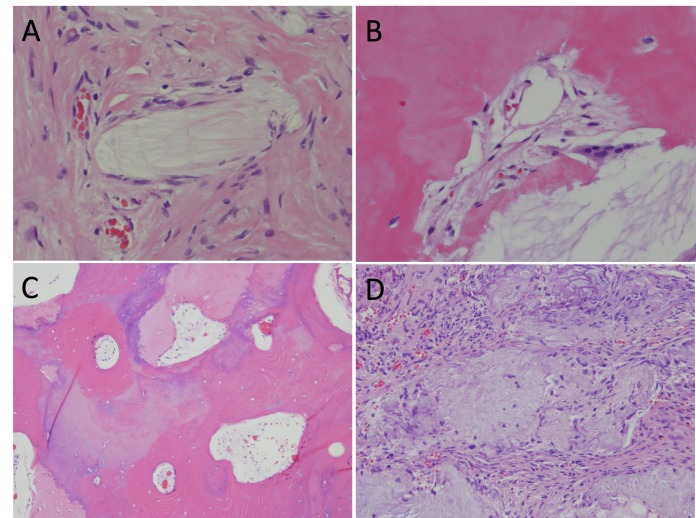
Photomicrographs of surgical specimens Photomicrographs of surgical specimens. Heterogeneous tissue containing nodules of chondromyxoid material with palisading spindle-to-epithelioid cells at the edges (A). Chondromyxoid matrix with coarse fibrillar and focal alveolar pattern with dystrophic calcifications and osseous metaplasia (B). Areas of immature and mature bone with trabeculae and lacunae containing osteocytes (C). Sparse chronic inflammatory cells in the fibrovascular stroma between chondromyxoid nodules (D).

The specimen consisted of heterogeneous tissue containing nodules of chondromyxoid material with palisading spindle-to-epithelioid cells at the edges. The chondromyxoid matrix was coarsely fibrillar and had a focal alveolar pattern with dystrophic calcifications and osseous metaplasia. Both immature and mature bone with trabeculae and lacunae that contained osteocytes were present. There were sparse chronic inflammatory cells in the fibrovascular stroma between chondromyxoid nodules (Figure 4).

Immunohistochemical staining was performed and revealed negative staining for S-100 protein, GFAP, and minimal MIB-1 labeling. The trichrome stain revealed a mesenchymal origin.

### Postoperative course

The clinical course following surgery was unremarkable. The patient was discharged home on postoperative day 5 with no changes in her neurological exam. At her four-month follow-up visit, the patient was clinically stable and remained seizure-free. Follow-up MRI showed postoperative changes along the resection bed without evidence of residual or recurrent tumor. The brainstem lesion showed no change or growth.

## Discussion

The first intracranial calcifying pseudoneoplasm was reported in 1978 by Rhodes and Davis when an unusual fibro-osseous component was identified in intracranial masses at autopsy and was described as an aberrant type of osseous metaplasia [4]. Since then, 40 intracranial lesions have been found [6].

These lesions are typically extra-axial and occur both infra- and supratentorially [1, 7]. The radiographic appearance on CT is nonspecific and consistent with a well-demarcated and calcified intracranial mass. Consequently, CAPNONs are frequently confused with other intracranial lesions, including cavernous malformations, meningiomas, malignant lesions, and infections, such as tuberculosis [6-7]. The appearance of CAPNONs on MRI has been described as a hypointensity on T1- and T2-weighted images with minimal linear internal or partial rim of contrast enhancement [7]. Surrounding brain edema (hyperintensity on T2) is very uncommon and may represent biological activity or tumor progression [2, 8]. In our patient, the presence of several intra-axial calcified lesions raised suspicion of multiple cavernous malformations, although genetic screening for KRIT-1 and CCM-2 mutations was negative. The progression of adjacent hyperintensity on T2-weighted images and worsening symptoms suggests that edema may represent a sign of increased tissue response to the tumor and that lesions with significant edema should be considered for surgical treatment.

The distinctive histological features of CAPNONs include 1) a chondromyxoid matrix in a nodular pattern; 2) palisading spindle to epithelioid cells; 3) variable amounts of fibrous stroma; 4) osseous structures containing birefringent lamellar bone, calcifications, psammoma bodies; and 5) foreign-body reaction with giant cells [7]. However, the presence of all these elements in a single lesion is highly variable, and based on previous reports, lesions might not show all of these features [7]. Moreover, the mitotic activity and cellular atypia of the palisading cells is typically minimal or absent [6], and immunohistochemical stains are positive for vimentin and epithelial membrane antigen (EMA) and negative for S-100 protein and glial fibrillary acidic protein (GFAP) [2]. The EMA reactivity may support an arachnoid cell origin, but the tissue of origin in CAPNONs is still in question. According to Bertoni, et al., calcifying pseudoneoplasms represent benign, nonneoplastic lesions that may be the result of unusual tumoral calcinosis [9]. In contrast, the most well-accepted theory proposes that CAPNONs arise throughout the CNS as a healing process following different types of injuries, including infection, trauma, or inflammation, which may account for the differences in histopathologic findings [7]. Rodriguez, et al. described an unusual case of low-grade cerebellar ependymoma contiguous with a CAPNON where the histopathologic analysis showed granulomatous inflammation and multinucleated giant cells in the vicinity of the ependymoma and piloid gliosis surrounding the calcified mass [10]. The authors postulated that CAPNONs result from a proliferative tumefactive process rather than bony metaplasia as Rhodes and Davis had suggested [4]. In comparison to the literature, the current report has a distinct manifestation as it showed multiple intracranial calcifying pseudoneoplasms and provided more information on the pathogenesis of these lesions. The distribution of these calcifying pseudoneoplasms in the infratentorial and supratentorial compartments suggests an underlying abnormal proliferative process that might have been initiated by the multifocal insults on the brain from previous brain abscesses. Although the diagnosis of multiple CAPNONs requires tissue diagnosis, the radiographic appearance of the lesions on CT and MRI in conjunction with the clinical circumstances suggests that our patient had multiple CAPNONs.

The prognosis following complete resection is typically favorable, and complications tend to arise from adhesions to the arachnoid overlying the cranial nerves, blood vessels, or brainstem, rendering complete excision unsafe [2]. In a series of 14 patients, Bertoni, et al. reported recurrence in one case after intralesional excision where the lesion extended widely in the posterior fossa and invaded multiple bones [9].

## Conclusions

Intracranial calcifying pseudoneoplasms are rare and can manifest as extra-axial or intra-axial masses. To our knowledge, the current report is the first to demonstrate the development of multiple intracranial CAPNONs and sheds more light on the origin of these lesions. Although the lack of specific radiographic features makes their diagnosis difficult, CAPNONs should be included in the differential diagnosis of single or multiple intracranial calcified lesions with hypointensity on T1- and T2-weighted MR images and minimal contrast enhancement. The pathogenesis of CAPNONs remains undefined, but the distinctive aspects presented in this case suggest that the eliciting factor is likely an abnormal reaction of the brain following an insult.
